# Estudio transversal de reportes de intento de suicidio en Colombia durante la pandemia del covid-19

**DOI:** 10.15446/rsap.V25n4.108002

**Published:** 2023-07-01

**Authors:** María Ana González-Alvarez, Viviana A. Rodríguez-Romero, Diana C. Acuña, Isabel del Socorro Moreno-Luna

**Affiliations:** 1 MG: MD. M. Sc. Epidemiología Clínica. M. Sc. Salud Internacional y Medicina Tropical. Departamento de Epidemiología Clínica y Bioestadística, Pontificia Universidad Javeriana. Bogotá, Colombia. m.gonzaleza@javeriana.edu.co Pontificia Universidad Javeriana Salud Internacional y Medicina Tropical Departamento de Epidemiología Clínica y Bioestadística Pontificia Universidad Javeriana Bogotá Colombia m.gonzaleza@javeriana.edu.co; 2 VR: Estad. M. Sc. Epidemiología Clínica Ph.D. Bioestadística. Departamento de Epidemiología Clínica y Bioestadística. Pontificia Universidad Javeriana. Bogotá, Colombia. viviana.rodriguez@javeriana.edu.co Pontificia Universidad Javeriana Bioestadística Departamento de Epidemiología Clínica y Bioestadística Pontificia Universidad Javeriana Bogotá Colombia viviana.rodriguez@javeriana.edu.co; 3 DA: ABG. M. Sc. Derechos Humanos. Facultad de Jurisprudencia, Universidad Colegio Mayor de Nuestra Señora del Rosario. Bogotá, Colombia. dianaca.acuna@urosario.edu.co Derechos Humanos Facultad de Jurisprudencia Universidad Colegio Mayor de Nuestra Señora del Rosario Bogotá Colombia dianaca.acuna@urosario.edu.co; 4 IM: PSC. M. Sc. Departamento de Epidemiología Clínica y Bioestadística. Pontificia Universidad Javeriana. Bogotá, Colombia. Pontificia Universidad Javeriana Departamento de Epidemiología Clínica y Bioestadística Pontificia Universidad Javeriana Bogotá Colombia

**Keywords:** Reportes, suicidio, pandemia, confinamiento, covid-19, salud mental, vigilancia epidemiológica *(fuente: DeCS, BIREME)*, Reports, suicide, pandemic, confinement, covid-19, mental health, epidemiological surveillance *(source: MeSH, NLM)*

## Abstract

**Objetivos:**

Caracterizar a la población que reporta casos de intento de suicidio durante el periodo de confinamiento y posconfinamiento inmediato en Colombia, según los reportes oficiales recolectados por el Sistema de Vigilancia Epidemiológica del Instituto Nacional de Salud (Sivigila) y determinar si existen patrones de presentación y variaciones con respecto a las características de la población que reportó casos en el año 2019.

**Métodos:**

Estudio observacional descriptivo de corte transversal seriado. Se hizo un análisis de correspondencia múltiple sobre las fichas de vigilancia epidemiológica de "intento de suicidio" recolectadas en Colombia desde el 25 de marzo hasta el 31 de diciembre del año 2020 y durante el mismo periodo de tiempo del año 2019.

**Resultados:**

Se analizó un total de 42 102 reportes de "intento de suicidio" (23 745 en 2019 y 18 357 en 2020) y se constató que hubo una reducción de aproximadamente 23% en el número de reportes en el año 2020, en comparación con el 2019. Se encontraron patrones de presentación, considerando las características sociodemográficas de los reportantes, pero no se hallaron diferencias entre los periodos anuales estudiados.

**Conclusiones:**

Si bien en el año 2020 hubo una reducción en el número de reportes oficiales recolectados de "intento de suicidio" no se puede afirmar que hubo una disminución efectiva de casos de este evento en el país.

## Abstract (en)

**Objective:**

To characterize the population that reports cases of attempted suicide during the confinement period and immediate post-confinement in Colombia according to the official reports collected by the epidemiological surveillance system of the National Institute of Health (Sivigila) and to determine if there are patterns of presentation and variations concerning the characteristics of the population that reported cases in 2019.

**Methods:**

A serial cross-sectional descriptive observational study and multiple correspondence analysis on epidemiological surveillance sheets of "suicide attempts" collected in Colombia from March 25 to December 31, 2020, and during the same period in 2019.

**Results:**

42,102 reports of "Attempted Suicide" were analyzed (23,745 in 2019 and 18,357 in 2020), and there was a reduction in approximately 23% of the number of reports in the year 2020 compared to the year 2019. In addition, patterns of presence were found considering the sociodemographic characteristics of the reporters, but no differences were identified between the annual periods studied.

**Conclusions:**

Although in the year 2020, there was a reduction in the number of official reports collected of "suicide attempts", it cannot be affirmed that there was an effective decrease in cases of this event in the country.

La crisis desencadenada por la pandemia de la enfermedad covid-19 generó cambios en múltiples esferas de la vida colectiva de la humanidad 1. Casi todos los países implementaron pautas de aislamiento de la población y en muchos de estos se decretaron diferentes confinamientos 1.

Paralelamente al curso de la pandemia, se sugirió la posible relación entre el aislamiento preventivo y el aumento de los intentos suicidas 2. Las hipótesis sobre el origen de este incremento apuntan a condiciones estructurales de base, las cuales se acentuaron durante la pandemia 2. Entre estas se cuenta la pobre relación del hombre moderno con la incertidumbre, la predisposición basal actual a desórdenes ansiosos o depresivos y el abuso de alcohol o de sustancias psicoactivas, entre otras 3,4. Adicionalmente, nuevos factores propios de esta crisis, como estresores ambientales, miedo al contagio, pérdidas de seres cercanos, disfunción familiar y desempleo se describieron como catalizadores de los intentos suicidas a escala mundial 5. Si bien existe una aceptación generalizada de la anterior afirmación, en Colombia no se ha publicado evidencia robusta que soporte este hecho.

El 25 de marzo del año 2020 inició el periodo conocido como "emergencia sanitaria" en Colombia y con este se instauraron los confinamientos y las restricciones sociales del primer año de la pandemia en el país 6. Se han publicado los reportes preliminares realizados durante el primer mes del confinamiento de aquel año y, contrariamente a la evidencia en otras regiones, los reportes de los intentos suicidas disminuyeron drásticamente en Colombia durante dicho periodo de cuatro semanas 7. El subregistro, la recolección de datos incompletos y las barreras de acceso al sistema de salud durante el confinamiento son las principales hipótesis que explican este hallazgo 7.

Aunque los intentos suicidas son eventos de notificación obligatoria para el seguimiento epidemiológico, aún hoy no existe un sistema unificado de reportes de estos eventos a nivel nacional 8. Una de las principales rutas para la recopilación de información se realiza a través del Instituto Nacional de Salud (INS) 9. La recolección de datos se lleva a cabo en todos los centros de salud del país por parte del personal clínico que atiende los casos y mediante el registro de la "ficha de notificación individual" 10. La información recolectada es agrupada y concentrada en el registro único del Sistema de Vigilancia en Salud Pública (Sivigila) del INS 7,11.

El objetivo principal de este trabajo es caracterizar a la población que fue objeto de reportes de casos de intento de suicidio durante el primer año de la pandemia en Colombia y determinar si existen variaciones con respecto a las características de la población del año 2019. Adicionalmente, se identificaron patrones de presentación (demográficos, sociales, etc.) de los reportes de los sujetos durante este periodo, con el objetivo de generar nuevas hipótesis sobre el comportamiento de este fenómeno durante el confinamiento.

## MATERIALES Y MÉTODOS

Se diseñó un estudio observacional descriptivo de corte transversal seriado, como análisis secundario de las fichas de vigilancia epidemiológica de "intento de suicidio" recolectados en Colombia por el INS desde el 25 de marzo hasta el 31 de diciembre del 2020 y durante el mismo periodo del 2019. Se incluyeron todos los reportes en el periodo de estudio.

El Departamento de Propiedad Intelectual del INS entregó las bases de datos del evento "intento de suicidio (evento 356)", en un acuerdo de transferencia de datos firmado en conjunto con la Pontificia Universidad Javeriana, sede Bogotá, Colombia. Las bases de datos fueron entregadas sin identificación y completamente anonimizadas.

Se presentan estadísticas descriptivas de todas las variables seleccionadas. Para determinar los patrones de presentación basados en las características sociodemográfica de los sujetos y los descriptores de los eventos, se hizo un análisis de correspondencia múltiple (ACM). Se llevó a cabo el análisis de forma independiente para los años 2019 y 2020 y la combinación de estos dos periodos para identificar diferencias en las características de cada año. Se utilizó el criterio del codo y máxima curvatura del gráfico de la varianza o Screeplot para retener las dimensiones 12.

Tanto la manipulación de datos como el análisis estadístico se llevaron a cabo en su totalidad en la plataforma RStudio 13 y se utilizaron los paquetes del *software* estadístico R "FactoMineR" 14 y "factoextra" 15 para llevar a cabo el ACM.

Esta investigación se enmarca dentro de la Declaración de Helsinki 16 y según las directrices de la Resolución 8430 de 1993 del Ministerio de Salud y Protección Social de Colombia; este trabajo se encuentra calificado como "investigación sin riesgo" 17. El protocolo de investigación fue aprobado por el Comité de Investigaciones y de Ética Institucional de la Facultad de Medicina de la Pontificia Universidad Javeriana en marzo del año 2021 (número de carta: FM-CIE-0190-21).

## RESULTADOS

**Figura 1:**
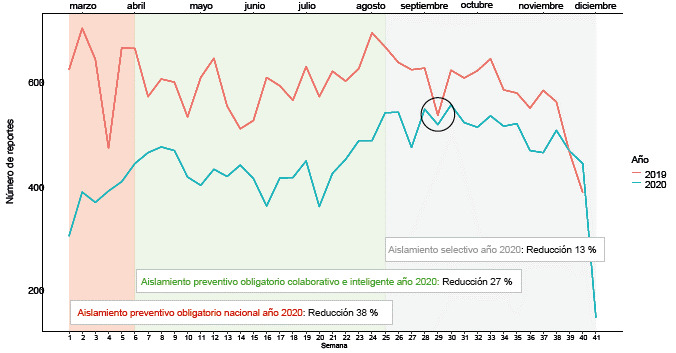
Serie de tiempo de los reportes de "intento de suicidio" para el periodo de estudio (25 de marzo al 31 de diciembre) en los años 2019 y 2020

Se registró un total de 42 102 reportes de "intento de suicidio" (23 745 en 2019 y 18 357 en 2020), con una reducción del 22,7% en el primer año de la pandemia comparado con el año anterior. En el periodo catalogado como "aislamiento preventivo obligatorio nacional" (25/03/20 - 27/04/20), la reducción en el número de reportes para el 2020 comparado con el año 20ig fue del 38%, mientras que para los periodos denominados como "aislamiento preventivo obligatorio colaborativo e inteligente" (28/04/20 - 31/08/20) y la "nueva normalidad" (0i/0g/20 - 31/12/20) esta reducción fue de 27% y 13%, respectivamente (Figura 1).

El periodo de las últimas dos semanas del mes de octubre del año 2020 (semanas 30 y 31 en la Figura 1) fue el único lapso de tiempo en el cual el número de los reportes del año 2020 superó a aquel del 201g. La descripción completa de las características de los sujetos recolectadas en las fichas para ambos años se encuentra en la Tabla 1. No hubo datos faltantes en las variables seleccionadas.

### Representación de las características sociodemográficas de las personas en los reportes de "intento de suicidio"

**Figura 2:**
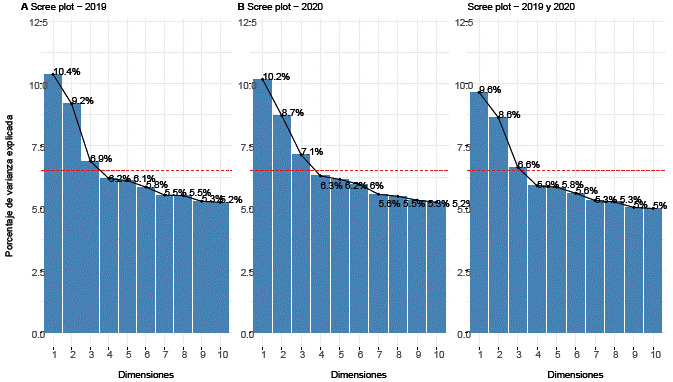
Porcentaje de varianza explicada por dimensiones para los registros de "intento de suicidio" para los años 2019, 2020 y para el conjunto de estos

Al realizar el análisis de correspondencia múltiple (ACM), la proporción de varianza retenida por las tres primeras dimensiones fue de poco más del 25% en cada periodo estudiado, es decir, que las dimensiones retenidas contribuyen a explicar solamente un cuarto del fenómeno de "intento de suicidio" (véase Figura 2). En este trabajo se decidió omitir la información contenida en la tercera dimensión, considerando la menor variabilidad retenida por esta; no obstante, su análisis puede ser consultado en el repositorio virtual de este trabajo 18.

**Figura 3:**
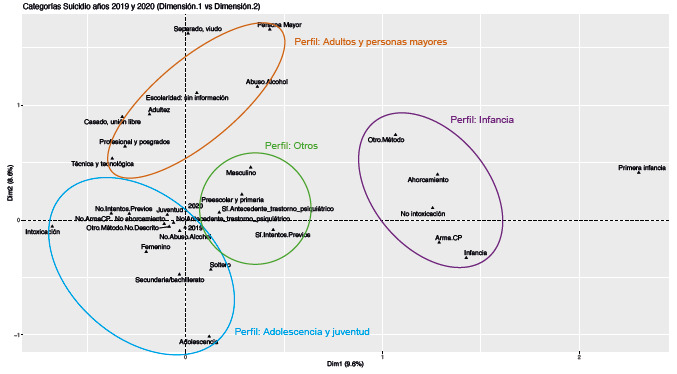
Características sociodemográficas de las personas en los reportes de "intento de suicidio" para el conjunto de años 2019 y 2020 (Dim. 1 vs. Dim. 2)

No se encontraron diferencias cualitativas relevantes en los periodos estudiados, razón por la cual únicamente se presenta el análisis de la combinación de los años 201g y 2020. La Figura 3 muestra los resultados del ACM para las dos primeras dimensiones y para el conjunto de los dos años estudiados. A continuación se describen los patrones identificados.

**Tabla 1:** Descripción de los reportes de "Intento de suicidio" para los años 2019 y 2020

Nombre	2019		2020	
	n	%	n	%
Total de reportes	23 747	100	18 357	100
Ciclo de vida				
Primera infancia (0 - 5 años)	9	< 0.1	12	< 0.1
Infancia (6 - 11 años )	598	2.5	340	1.9
Adolescencia (12 - 18 años)	8372	35.3	5740	31.3
Juventud (19 - 26 años)	6983	29.4	5747	31.3
Adultez (27 - 59 años)	7198	30.3	5933	32.3
Persona mayor (60 años y más )	587	2.5	585	3.2
Sexo				
Femenino	15 050	63.4	11 192	61.0
Masculino	8697	36.6	7165	39.0
Intentos previos	9252	38.9	7218	39.3
Antecedente de trastorno psiquiátrico	5998	25.2	5060	27.6
Abuso o dependencia del alcohol	1 773	7.4	1 410	7.7
Estado civil				
Soltero	16 399	69.1	12 751	69.5
Casado, unión libre	6620	27.9	5060	27.6
Separado, viudo	728	3.1	546	3.0
Escolaridad				
Ninguno/sin información	1871	7.9	1641	8.9
Preescolar y primaria	5751	24.2	4444	24.2
Secundaria/bachillerato	12163	51.2	9250	50.4
Técnica y tecnológica	2635	11.1	1977	10.8
Profesional y posgrados	1326	5.6	1045	5.7
Mecanismo de lesión (*)				
Intoxicación	15 416	65.0	11 934	65.0
Ahorcamiento/Asfixia	1780	7.5	1509	8.2
Lesión por arma cortopunzante	5430	22.9	4113	22.4
Otro	1752	7.4	1287	7.0

Respuesta del 100 % en todos los apartes (^*^) Variables no mutuamente excluyentes

### Perfil "Intento de suicidio en la infancia"

La categoría de los infantes (de los cinco a los once años) se encuentra lejana al intercepto y, por tanto, bien representada en el análisis. En este perfil se encuentran las categorías de mecanismos de lesión como "ahorcamiento", "lesión por arma corto punzante", "no intoxicación" y "otros métodos" (véase Figura 3).

### Perfil "intento de suicidio en adolescentes y en jóvenes"

Dentro de esta agrupación existente dos conglomerados que se encuentran relativamente distantes en las coordenadas del mapa. El primero es el grupo de los adolescentes (12 a 18 años) que se relaciona con las categorías de nivel educativo de "secundaria y bachillerato". Adicionalmente, este perfil se encuentra conformado por la clasificación de "soltero" como estado civil y sexo "femenino" (véase Figura 3). Según esta información, las mujeres adolescentes que intentan suicidarse en su mayoría se encuentran escolarizadas, cursando los últimos niveles de la secundaria, y son solteras. No se observa relación con abuso de alcohol ni con los distintos tipos de mecanismos descritos.

El segundo perfil hace referencia a los adultos jóvenes (1g-27 años) y contiene los distintos tipos de mecanismos de lesión utilizados por los sujetos que no han tenido un intento previo. En este conjunto se encuentra que la categoría del primer intento suicida ("no intentos previos") se relaciona con "intoxicación" y con otros mecanismos de lesión no descritos en los registros de vigilancia epidemiológica como mecanismos causales "otros" (véase Figura 3).

### Perfil "intento de suicidio en adultos y en personas mayores"

Localizados en el mapa de dispersión opuesto al grupo de los "adolescentes", se encuentran los registros de "personas mayores" cercano a la categoría de estado civil "separado, viudo", con antecedente de "abuso de alcohol" y sin información sobre la escolaridad (Figura 3). Cerca de estos se encuentran las categorías de "adultez", "casado, unión libre" y "técnicas y tecnológicas", "profesionales y posgrados" (Figura 3).

### Perfil "intento de suicidio y otros"

La categoría de "preescolar y primaria" está cercana a la categoría de sexo "masculino", "antecedente de trastorno psiquiátrico", "antecedente de intentos suicidas previos" y "no abuso de alcohol". Sugerimos que este perfil contiene a los adultos que tienen una escolaridad máxima de "preescolar y primaria" (Figura 3).

El perfil anterior y aquel del grupo de los "jóvenes" se encuentran cercanos entre sí, como también cercanos al intercepto, razón por la cual puede que sean fenómenos que comparten características, y sus interpretaciones deben ser evaluadas con cautela por aportar poca variabilidad para explicar el fenómeno (véase Figura 3).

## DISCUSIÓN

En este estudio encontramos una reducción en el número de reportes oficiales al INS de aproximadamente el 23% durante los meses de marzo a diciembre del año 2020, en comparación con el mismo periodo del año anterior.

Los perfiles hallados en el ACM no se modificaron entre el 201g y el 2020 y esto aporta evidencia de que el fenómeno, por lo menos durante el primer año de la pandemia, se mantuvo cualitativamente estable y no cambió en sus características globales por el aislamiento o las medidas de control de la pandemia.

En apoyo de esta idea, Gerster publicó recientemente una serie de tiempo de los reportes oficiales de intentos de suicidio según información de la Policía Nacional del Ecuador desde el año 2015 hasta junio del 2021 19. Luego de analizar la información recolectada en 7 487 reportes, no se encontró evidencia que demostrara que las tasas de suicidio fueran más altas durante los 16 meses estudiados de la pandemia contra los años previos (RR 95% 0,97, IC: 0,02 - 1,02). No obstante, el grupo de investigación es cauteloso en interpretar sus resultados, teniendo en cuenta las restricciones de movilidad por la pandemia 19.

Esta restricción de la libre circulación y la ampliación de las barreras de acceso al sistema de salud pueden explicar la reducción en el número de reportes oficiales para el caso de intento de suicidio. Si bien desde el inicio de la pandemia se alertó sobre el impacto que esta tendría en la salud mental de la población, los sistemas de salud se volcaron completamente a lo derivado a la infección por el virus SARS-CoV-2 20.

Lo encontrado por Williams 21 en un estudio de cohorte retrospectivo en una comunidad urbana desfavorecida en el Reino Unido sustenta la anterior afirmación. Durante los tres primeros meses de la pandemia, las consultas por síntomas relacionados con salud mental en la región tuvieron una reducción del 50% de lo esperado (IC 95% 41,1-56,9), comparado con los años previos 21. Los autores concluyen que si bien esta disminución en las consultas de atención primaria en salud mental pudo hacerse evidente al inicio de la pandemia, sus efectos colaterales y alza subsecuente deben ser cuantificados en los meses e incluso años después de los confinamientos 21.

No podemos afirmar que menos reportes oficiales sea un indicativo de menos casos efectivos de intentos suicidas en el país, sugerimos que es factible que la información de reportes oficiales enmascare la verdadera dimensión del fenómeno. Los reportes del INS recogen la información tanto de los casos de suicidios consumados como de intentos suicidas y estos son contados igual. Por el contrario, el Instituto de Nacional de Medicina Legal de Colombia contabiliza diferencialmente los casos efectivos de muertes por causa de suicidio y las lesiones por intentos no consumados. Según reportes oficiales de esta entidad, las tasas anuales de muertes por suicidios en todo el territorio nacional fueron de 5,8 vs. 5,2 casos por 100 000 habitantes para el 2019 y el 2020, respectivamente, resultado sin mayor variación interanual 22. Por ende, las cifras de los canales de teleasesoría son datos útiles para ampliar la descripción del fenómeno durante la pandemia. Es aquí donde cobra mayor valor cotejar la información oficial encontrada con otras estrategias de recolección de datos para confrontar las conclusiones oficiales con investigaciones similares y contra diferentes fuentes de reportes.

Según un estudio de Brülhart 23, donde se analizó información procedente de ocho millones de llamadas telefónicas de teleasesoría de 19 países durante las primeras seis semanas de pandemia, el pico de consultas por este canal se alcanzó seis semanas luego de inicio de la pandemia, con 35% más llamadas que en niveles prepandémicos (IC 22,6 - 48,3) 23. La principal causa de consulta fue clasificada como "miedo y soledad", sin embargo, no se encontró un mayor número de llamadas a las líneas por casos específicos de ideación suicida, en comparación con años previos 23.

Remarcamos, considerando las limitaciones propias del diseño del estudio, que la naturaleza observacional descriptiva y la limitación del seguimiento temporal propio de este trabajo pueden llevar a conclusiones restringidas. Al analizar solamente ocho meses en dos periodos anuales seguidos, somos conscientes que información relevante previa a los confinamientos, y más importante aún, posterior a estos, no está siendo analizada en el presente estudio. Sugerimos que sea factible que el verdadero aumento del número de casos se empiece a hacer evidente en el posconfinamiento y con la instauración franca de la crisis socioeconómica de la pospandemia.

En apoyo de esta idea, un grupo de investigación de la Universidad de Hong Kong estudió las tendencias de muertes por suicidio antes, durante y luego de la crisis económica del año 2008. Los autores encontraron que en el año posterior al inicio de la crisis hubo un aumento en las tasas de suicidio de hombres de países de las Américas (4,2%) y de Europa (6,4%), comparado con los años previos, incluido el primer año de crisis (2000-2008) (24). Los autores sugieren que el aumento de los casos de suicidio se acentúa luego del inicio de las crisis globales 24.

En nuestros hallazgos encontramos que el grupo de los niños contiene mecanismos de lesión como "ahorcamiento" y "lesión por arma cortopunzante", los cuales sugieren la naturaleza doméstica del suceso y la disposición de los métodos para llevarlo a cabo. Los resultados de Kolves 25 en el año 2016 confirman nuestro hallazgo: luego de analizar la información sobre suicidios en niños a partir de la base de datos de mortalidad de la OMS y del Banco Mundial, concluyó que el principal método suicida de este grupo etario es el ahorcamiento, posiblemente relacionado con el fácil acceso al método 25.

Este hallazgo puede ser una bandera roja a nivel nacional sobre la aparición de un fenómeno de salud pública global relacionado con el aumento de intento de suicidios en niños y niñas prepúberes en la era digital actual 26. En apoyo de esta idea, Arnon 26 publicó en 2022 una encuesta de una muestra de niños estadounidenses entre 10 y 13 años, donde encontró qué haber experimentado "cyber matoneo" aumentaba el riesgo de ideación e intentos suicidas (OR 4,2; IC 95% 3,5-5,1) 26. Sugerimos explorar la probable asociación entre el "cyber matoneo" y los intentos suicidas en la población de niños colombianos en futuros estudios.

La ausencia de más descriptores en el grupo de "adolescentes" de nuestro análisis sugiere que hay información que no está siendo recolectada y es clave para explicar el fenómeno. Un ejemplo de las variables posibles para medir son el embarazo en adolescentes, la composición familiar, la urbanidad/ruralidad, la exposición a violencia doméstica, el estrés académico, la presión social y el matoneo escolar 27. Ninguna de estas variables está siendo sistemáticamente recolectada por el personal clínico que atiende los casos, a pesar de que muchas de estas hacen parte de las fichas sistema de vigilancia epidemiológica nacional.

En cuanto al perfil de los jóvenes, encontramos el mecanismo de lesión de "intoxicación" y dentro de esta categoría se agrupan todo tipo de envenenamientos medicamentosos, con pesticidas y fármacos veterinarios, con sustancias corrosivas y con productos de aseo. Este hallazgo es coherente con lo descrito a nivel global donde, y citando nuevamente la revisión de Kolves, el segundo mecanismo más frecuente para cometer actos suicidas en personas de 19 a 25 años es la intoxicación 25.

Por otro lado, y relacionado con el perfil de las personas mayores, la presencia de la categoría "separado, viudo" puede sugerir distintos tipos de soledad y de aislamiento asociados a la pérdida de redes de apoyo en el transcurso de la vida. Según las previsiones al inicio de los confinamientos de un grupo de investigación de la Universidad de Montpellier, Francia, la desconexión social y las rupturas con las redes de apoyo durante la pandemia podrían cobrar un peso inmenso en el crecimiento de los casos de intento de suicidio a nivel mundial 3,4.

La presencia de la categoría "consumo de alcohol" sugiere la posible relación de conductas disfuncionales como el alcoholismo con los intentos suicidas en este grupo etario. En apoyo de esta idea, Anestis 28 publicó una revisión sistemática en el año 2014, en la cual concluyó que aproximadamente un tercio de los sujetos que cometen suicidio a nivel global tienen niveles cuantificables de alcohol en sangre 28. Sugerimos estudiar en futuras investigaciones el aumento del consumo de alcohol en la población colombiana durante la pandemia y su posible relación con el aumento de la ideación suicida.

Por último, las categorías contenidas en el perfil de "intento de suicidio y otros" hablan sobre la relación existente y vastamente descrita entre enfermedad mental y riesgo suicida ("Sí antecedente de trastorno psiquiátrico", "Sí intentos previos") 29. Nuestra hipótesis es que este perfil representa a los hombres que no finalizaron el bachillerato y por ende no tuvieron acceso a más niveles educativos, con antecedente de enfermedades mentales e intentos de suicidio previos, y por ende sugiere una posible falta crónica de acceso a los cuidados y el tratamiento de salud mental en general, incluso previamente al inicio de la pandemia por covid-19.

Los modelos de ACM en el el caso de los reportes de "intento de suicidio" presentaron poca variabilidad explicada (cerca al 25% con tres dimensiones retenidas). Este hallazgo sugiere que puede que existan factores de riesgo y variables que expliquen y describan el fenómeno que no estén siendo consideradas en las fichas de vigilancia epidemiológica.

Una de las variables que cobran más relevancia al hablar de bienestar mental e intentos de suicidio es la composición y el funcionamiento familiar. Una de nuestras primeras recomendaciones a los sistemas de vigilancia es considerar herramientas validadas como el cuestionario del Apgar familiar 30 para determinar la percepción de quienes reportan casos de violencia en relación con el funcionamiento de las familias y apreciar su satisfacción con las principales redes de apoyo 30. Incluir cuestionarios como el Apgar familiar y entrenar al personal clínico del nivel primario de salud en su correcto uso y diligenciamiento puede ser una estrategia relativamente fácil y práctica para entender más sobre los fenómenos de las violencias y quienes las padecen 30.

Reconocemos que todos los contextos violentos pueden ser desencadenantes de intentos suicidas 27. Por esta razón y teniendo en cuenta que el INS también recopila información sobre violencia de género e intrafamiliar con la ficha por evento INS: 875 31, recomendamos cruzar esta información con aquella recogida en la ficha de "intentos suicidas" para ampliar el estudio de la sinergia de estos fenómenos y desarrollar estrategias de prevención más integrales.

Con respecto a nuestro periodo de estudio, reconocemos que estudiar únicamente dos años consecutivos y centrarnos solamente en el primer año de la pandemia puede llevarnos a observaciones incompletas y fragmentadas. Reconociendo esta limitante, proponemos analizar las tendencias y frecuencias del número de reportes oficiales del fenómeno en un rango de tiempo mucho más amplio.

Por último y considerando lo anterior, cobra más relevancia la cooperación y la integración de nuevos equipos transdisciplinarios de investigación, diferentes y variados métodos de recolección de datos, la utilización de métodos cualitativos, las nuevas estrategias de análisis y una interpretación integral y holística de un fenómeno complejo como los son los intentos de suicidio en el país ♠
